# Critical Convex‐Type ST Elevation Correlate With Ventricular Tachyarrhythmia in Takotsubo Cardiomyopathy

**DOI:** 10.1002/clc.70056

**Published:** 2024-12-09

**Authors:** Jen‐Te Hsu, Ju‐Feng Hsiao, See‐Khong Chin, Yu‐Cheng Hsu, Meng‐Huan Lei

**Affiliations:** ^1^ Department of Internal Medicine, Divisions of Cardiology Lo‐Hsu Medical Foundation Lotung Poh‐Ai Hospital Yilan County Taiwan

**Keywords:** convex‐type ST elevation, lambda‐wave ST elevation, takotsubo cardiomyopathy, tombstoning ST elevation, ventricular tachyarrhythmia

## Abstract

**Background:**

Ventricular tachyarrhythmia (VT) occasionally occurred in patients with Takotsubo cardiomyopathy (TC). Two convex‐type ST elevations were significantly related to VT in coronary artery disease.

**Methods:**

This study assessed the correlation between VT and critical ECG patterns, as well other independent predictive factors of in‐hospital outcome. Fifty‐five consecutive patients fulfilled the diagnostic criteria of Takotsubo Italian Network (TIN) were retrospectively enrolled. The patients were classified into two groups according to their critical ECG patterns and VT occurrence. In‐hospital outcomes and influencing factors were analyzed.

**Results:**

The incidence of VT was higher in the critical ECG group than in the Noncritical ECG group (43.8% vs. 2.6%, *p* < 0.001). In‐hospital death was more common in the critical ECG group than in the Noncritical ECG group (25.0% vs. 5.1%, *p* = 0.032). The composite end‐point (combined VT and in‐hospital death) revealed significant differences between these two groups (50.0% vs 7.7%, *p* < 0.001). Multi‐variate analysis proved critical ECG type as one independent risk factor of VT (odds ratio [OR] = 61.8, *p* = 0.009) and the composite end‐point (OR = 12.4, *p* = 0.007). The prolong QRS width ( ≥ 105 ms) was another independent factor for predicting VT (OR = 1.06, *p* = 0.022) and composite end‐point (OR = 1.05, *p* = 0.017).

**Conclusions:**

Critical ECG types including tombstoning ST elevation and lambda‐wave ST elevation have strong impact on short‐term outcomes. Additionally, conduction disturbance with prolong QRS ≥ 105 ms also has independent predicting role for poor prognosis.

## Summary

- Key findings
  - Both critical ECG types and prolong QRS 105 ms had independent predicting role for poor prognosis in patients with stress‐induced cardiomyopathy.
- What is known and what is new?
  - An lambda‐wave ST elevation and tombstoning ST elevation may predict malignant arrhythmias in patients with CAD and STEMI.
  - For patients with stress‐induced cardiomyopathy, the critical ECG types including tombstoning ST elevation and lambda‐wave ST elevation had strong impact for VT and in‐hospital death.
- What is the implication, and what should change now?
  - Above abnormal ECG signs identifying high‐risk patients and implementing close monitoring and aggressive interventions.

## Graphical abstract

Ventricular tachyarrhythmia (VT) occasionally occurred in patients with Takotsubo cardiomyopathy (TC). This study assessed the correlation between VT and critical ECG patterns. Critical ECG types including tombstoning ST elevation and lambda‐wave ST elevation have strong impact on short‐term outcomes.

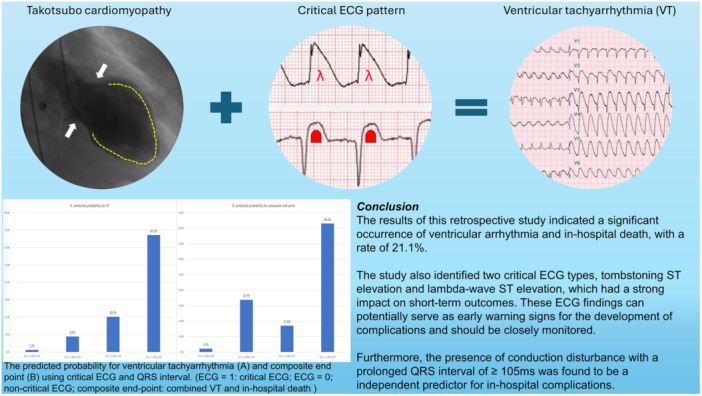

## 1 Introduction

Ventricular tachyarrhythmia (VT), which include ventricular tachycardia and ventricular fibrillation (VF), is the most common cause of coronary artery disease (CAD)‐induced sudden cardiac death.

Two extre convex‐type ST elevation patterns are strongly related to VT.

A notched, giant J‐wave accompanied by a steeply down‐sloping ST‐T segment elevation and a negative T wave is defined as a lambda‐like waveform, which was labeled by Gussak et al. [1] Lambda‐wave ST elevation may predict malignant arrhythmias in patients with CAD [2].

Tombstoning ST elevation was observed in one fourth of patients with ST‐elevation myocardial infarction (STEMI). This ECG abnormality is associated with an increased mortality rate, higher incidence of heart failure and VF and decreased left ventricular ejection fraction [3, 4, 5].

These two critical ECG patterns have been previously observed in patients with stress‐induced cardiomyopathy at our hospital.

This study aimed to identify the critical ECG features that may affect the short‐term prognosis of patients with takotsubo cardiomyopathy (TC).

## 2 Methods

### 2.1 Study Population

The study population consisted of 55 consecutive patients diagnosed with Takotsubo cardiomyopathy and fulfilled the diagnostic criteria of the Takotsubo Italian Network (TIN) [6]. The patients were selected from the Department of Cardiology at Poh‐Ai Hospital between April 2010 and March 2022. All patients provided written informed consent and the study was approved by the local ethical committee.

### 2.2 Inclusion Criteria

**Figure 1:**
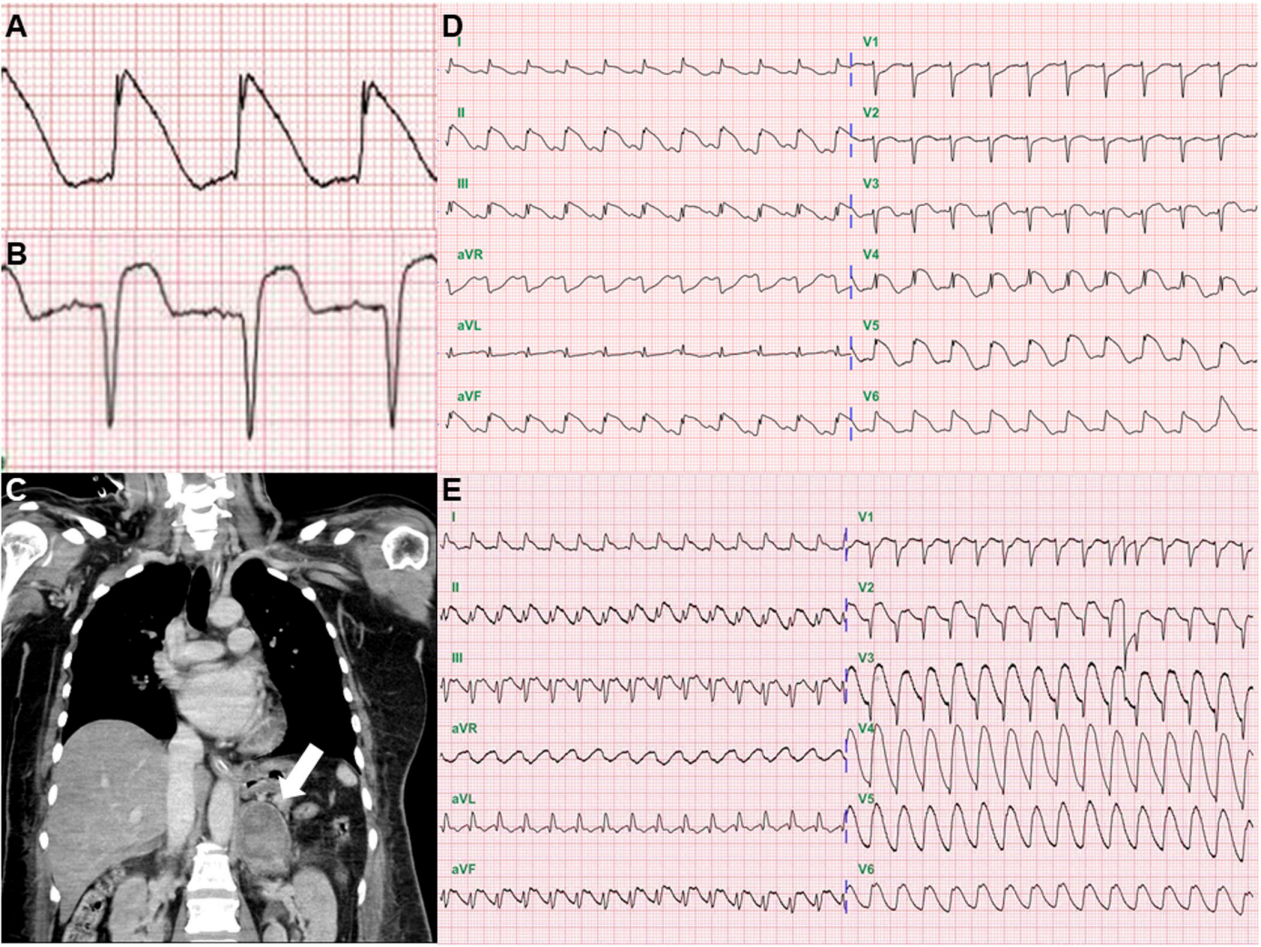
(A) lambda‐wave ST elevation; (B) tombstoning ST elevation; (C) an enhanced abdominal computed tomographic scan showed a large (3.9 × 5.2 cm) left adrenal mass (arrow) with areas of heterogeneous high density and infiltrative margin; (D) initial ECG revealed lambda‐wave ST elevation; (E) monomorphic VT attack.

The lambda‐wave ST elevation pattern was defined as the presence of an elevated J wave (amplitude ≥ 1/4 R wave and ≤ R wave) followed by a steep down‐sloping elevated ST segment that merges with the inverted T wave [7]. (Figure 1A)

Tombstoning ST elevation was defined as follows [8, 9]: (Figure 1B)

1. No R waves or significantly reduced R wave amplitude, less than 40 ms duration, and no waves beneath the isoelectric line following the R wave.
2. The convex ST segment is blurred with the descending arm of the R‐wave or the ascending arm of the QRS/QR complex.
3. Peak of the convex ST segment higher than the R wave amplitude.
4. The convex ST segment is blurred with the ascending arm of the T‐wave.

Patients who exhibited these ECG patterns in at least two contiguous leads were included in the critical ECG group and compared with other patients in the study. All critical ECGs were obtained within the first 3 days of admission, including the initial examination at the ER and serial follow‐ups at the ICU and ward. All patients accepted continuous ECG monitoring during their hospitalization. All patients underwent a clinical examination and echocardiography, and their age, sex, medical history, and stressors were recorded. Stressors were categorized as uncertain, emotional, or medical. Additionally, all patients underwent coronary angiography to document the coronary artery and left ventriculogram.

### 2.3 Follow‐Up and Definition of Outcome

In‐hospital complications were recorded as VT, ventricular fibrillation, all‐cause death, cardiogenic arrest, pulmonary edema, stroke, new atrial fibrillation, ventilator usage, and major bleeding (hemoglobin reduction ≥ 2 g/dL) [10].

All patients were continuously monitored by bedside ECG monitors or wireless remote ECG monitors since admission. All documented VT episodes were classified as sustained VT.

### 2.4 Statistical Analysis

Continuous variables of normal distribution are presented as the mean ± standard deviation. The statistical significance of differences between such variables was analyzed using Student's *t*‐test. Categorical variables were expressed as proportions and compared using the chi‐square test. Binary logistic regression analysis was used to calculate the odds ratio (OR) and 95% confidence interval (CI) of independent risk factors and propensity scores. Statistical significance was set at *p* < 0.05. The cut‐off value was derived using ROC curve method.

## 3 Results

### 3.1 Critical ECG Related Clinical Events

All patients were first classified into critical ECG group versus Noncritical ECG group to analyze major clinical events (Table 1). The incidence of VT was higher in the critical ECG group than in the Noncritical ECG group (43.8% vs. 2.6%, *p* < 0.001). In addition, in‐hospital death was more common in the critical ECG group than in the Noncritical ECG group (25.0% vs. 5.1%, *p* = 0.032). The composite end‐point (combined VT and in‐hospital death) revealed significant differences between these two groups (50.0% vs 7.7%, *p* < 0.001). The incidence of mechanical ventilation usage showed a strong trend in the critical ECG group versus the Noncritical ECG group (50.0% vs. 23.1%, *p* = 0.050). Other clinical events including cardiac arrest, pulmonary edema, atrial fibrillation and major bleeding were not significantly different between the two groups.

**Table 1:** Clinical events between critical ECG versus Noncritical ECG groups.

Parameter	Critical ECG (*n* = 16)	Noncritical ECG (*n* = 39)	*P* value
VT	7/16 (43.8%)	1/39 (2.6%)	< 0.001
In‐hospital Death	4/16 (25.0%)	2/39 (5.1%)	0.032
Ventilator usage	8/16 (50.0%)	9/39 (23.1%)	0.050
Pulmonary edema	4/16 (25.0%)	4/39 (10.3%)	0.159
Atrial fibrillation	2/16 (12.5%)	2/39 (5.1%)	0.339
Cardiac arrest	2/16 (12.5%)	1/39 (2.6%)	0.141
Major bleeding	1/16 (6.3%)	0/39 (0.0%)	0.115
Stroke	0/16 (0.0%)	1/39 (2.6%)	0.518
Composite end‐point	8/16 (50.0%)	3/39 (7.7%)	< 0.001

*Note:* Composite end‐point = combined VT and in‐hospital death

Abbreviations: ECG, Electrocardiography; VF, ventricular fibrillation; VT, ventricular tachycardia.

The detailed characteristics of the 11 patients with VT or in‐hospital death are listed in Table 2.

**Table 2:** Detail characteristics of patients with composite end point.

Patient	ECG characteristics	Stress	Major cardiac event	Cardioversion
83 y/o male	Lambda‐type STE	Right femoral neck fracture	Monomophic VT followed with VF and in‐hospital death	No
81 y/o female	Lambda‐type STE	Urosepsis	Monomorphic VT and in‐hospital death	Yes
71 y/o female	Lambda‐type STE	Acute cholecystitis	Monomorphic VT	No
53 y/o female	Lambda‐type STE	pheochromocytoma	Monomorphic VT	Yes
64 y/o female	Lambda‐type STE	Head contusion	Monomorphic VT	Yes
54 y/o female	Lambda‐type STE	Urosepsis	Monomorphic VT, major bleeding (thrombocytopenia and ECMO wound oozing)	No
83 y/o male	Lambda‐type STE	Falling down injury	In‐hospital death	Yes
41 y/o female	Tombstone‐type STE	Uncertain	Monomorphic VT and in‐hospital death	Yes
50 y/o female	T inversion	Uncertain	Monomorphic VT	No
81 y/o female	Regular STE	Intracranial hemorrhage	In‐hospital death	No
82 y/o male	Regular STE	Pneumonia with sepsis	In‐hospital death	No

Abbreviations: ECMO, extracorporeal membrane oxygenation; STE, ST elevation; VF, ventricular fibrillation; VT, ventricular tachycardia.

Eight patients initially experienced monomorphic VT, and three died in‐hospital. Another three cases suffered from physical stress and in‐hospital death without documented VT. Lambda‐wave ST elevation was recorded in seven patients and tombstoning ST elevation occurred in one patient. Patients with these two critical ECG patterns have a high prevalence (50%) of achieving the composite end‐points. ST elevation without critical ECG patterns was noted in two patients who died in‐hospital. One patient had documented T inversion before the VT attack. Among all patients with composite endpoint, nine patients had physical stress as the preceding cause, and the exact etiology could not be documented in two patients by reviewing their medical records.

A 53 female patient with pheochromocytoma had an obvious lambda‐wave ST elevation before a VT attack. (Figure 1C‐E).

### 3.2 Patients' Characteristics

All patients were re‐classified according to the VT event for further analysis (Table 3).

**Table 3:** Clinical characteristics between VT and non‐VT groups.

Parameter	VT (*n* = 8)	Non‐VT (*n* = 47)	*P*
Age	62.1 ± 15.2	71.8 ± 13.7	0.074
BMI	24.4 ± 2.9	22.8 ± 6.0	0.490
Female	7/8 (87.5%)	32/47 (68.1%)	0.264
Diabetes mellitus	2/8 (25.0%)	15/47 (31.9%)	0.696
hypertension	6/8 (75.0%)	26/47 (55.3%)	0.297
CAD	1/8 (12.5%)	8/47 (17.0%)	0.749
PCI history	0/8 (0.0%)	3/47 (6.4%)	0.462
ESRD	0/8 (0.0%)	3/47 (6.4%)	0.462
eGFR (mL/min/1.73 m^2^)	70.1 ± 46.8	80.7 ± 48.1	0.567
COPD	2/8 (25.0%)	5/47 (10.6%)	0.876
Smoking	2/7 (28.6%)	4/47 (8.5%)	0.167
BNP (pg/mL)	1232.2 ± 1849.6	1401.6 ± 1395.6	0.794
Peak Troponin T	1899.8 ± 2003.6	899.9 ± 1469.8	0.088
Peak CPK (U/L)	1203.1 ± 1205.5	764.7 ± 991.8	0.275
Peak CK‐MB (U/L)	211.8 ± 357.9	811.7 ± 1025.9	0.287
LVEF (%)	31.7 ± 25.2	45.7 ± 28.6	0.312
Uncertain stress	3/8 (37.5%)	32/47 (68.1%)	0.071
Physical stress	5/8 (62.5%)	11/47 (23.4%)	
Emotional stress	0/8 (0.0%)	4/47 (8.5%)	
Hospitalization days	18.9 ± 21.0	13.0 ± 14.9	0.340
Medication			
Amiodarone	5/8 (62.5%)	7/47 (14.9%)	0.003
Dopamine	6/8 (75.0%)	5/47 (10.6%)	< 0.001
ACEI or ARB	1/8 (12.5%)	20/47 (42.6%)	0.106
Antiplatelet	7/8 (87.5%)	34/47 (72.3%)	0.363
Beta‐blocker	6/8 (75.0%)	23/47 (48.9%)	0.172
ECG characteristics			
Heart rate (bpm)	101.5 ± 18.8	88.9 ± 25.6	0.189
Critical ECG	7/8 (87.5%)	9/47 (19.1%)	< 0.001
QRS interval (msec)	115.0 ± 17.4	91.7 ± 23.7	0.011
QTc (msec)	436.4 ± 27.5	436.0 ± 43.5	0.981
J point elevation (mm)	7.3 ± 6.9	1.8 ± 2.3	0.061
Sum of STD (mm)	25.4 ± 23.1	6.4 ± 6.7	0.053
No of STD leads	4.6 ± 2.2	4.3 ± 1.5	0.700
Max T wave inversion (mm)	0.6 ± 0.9	1.9 ± 3.6	0.049

Abbreviations: AECG, electrocardiography; ARB, angiotensin receptor blocker; Antiplatelet, aspirin, clopidogrel, prasugrel, ticagrelor; BMI, body mass index; BNP, B‐type natriuretic peptide; CAD, coronary artery disease; CEI, angiotensin‐converting enzyme inhibitor; CK‐MB, creatine kinase‐MB; COPD, chronic obstructive pulmonary disease; CPK, creatine‐phospho‐kinase; eGFR, estimated glomerular filtration rate; ESRD, end stage renal disease; LVEF, left ventricular ejection fraction; PCI, percutaneous coronary intervention; QTc, corrected QT interval; STD, ST‐segment deviation; VT, ventricular tachyarrhythmia.

Eight patients (14.5%) experience in‐hospital VT attacks was included in the VT group. The other 47 patients were classified as no‐VT group. There were no statistically significant differences among baseline characteristics including age, sex, body mass index, diabetes, hypertension, end stage renal disease, estimated glomerular filtration rate, chronic obstructive pulmonary disease, smoking history, B‐type natriuretic peptide, peak cardiac enzyme, left ventricular ejection fraction (LVEF), hospitalization duration and stress sources.

The anti‐arrhythmia medication (amiodarone) usage rate was significantly higher in the VT group compared to the non‐VT group (62.5% vs. 14.9%, *p* = 0.003). Additionally, dopamine was prescribed more frequently in the VT group than in the non‐VT group (75.0% vs. 10.6%, *p* < 0.001).

The other medications, including angiotensin‐converting enzyme inhibitor, angiotensin receptor blocker, antiplatelet, beta‐blocker and statin, showed no significant difference in usage between the VT and non‐VT groups.

### 3.3 Electrocardiographic Features

Table 3 shows that the prevalence of critical ECG findings was significantly higher in the VT group than in the non‐VT group (87.5% vs. 19.1%, *p* < 0.001). The QRS width was significantly longer in the VT group than in the non‐VT group (115.0 ± 17.4 ms vs. 91.7 ± 23.7 ms, *p* = 0.011). The depth of maximal T inversion was less deep in the VT group than in the non‐VT group (0.6 ± 0.9 mm vs. 1.9 ± 3.6 mm, *p* = 0.049). The other ECG characteristics did not show significant difference between these two groups, including maximal J elevation, QT interval, sum of ST segment deviations and numbers of ST deviation leads. The significant parameters, including critical ECG finding, QRS width and maximal T inversion, were further analyzed by multivariate analysis. Multivariate analysis revealed that critical ECG pattern was an independent risk factor for VT (OR = 61.8, CI = 2.83–1348.55, *p* = 0.009) and the composite end‐point (OR = 12.4, CI = 2.0–76.33, *p* = 0.007). QRS width was also identified as an independent predictor of predicting VT (OR = 1.06, CI = 1.01–1.11, *p* = 0.022) and the composite end‐point (OR = 1.05, CI = 1.01–1.08, *p* = 0.017).

### 3.4 Propensity Score and Best Cut‐Off Value of QRS Width

**Figure 2:**
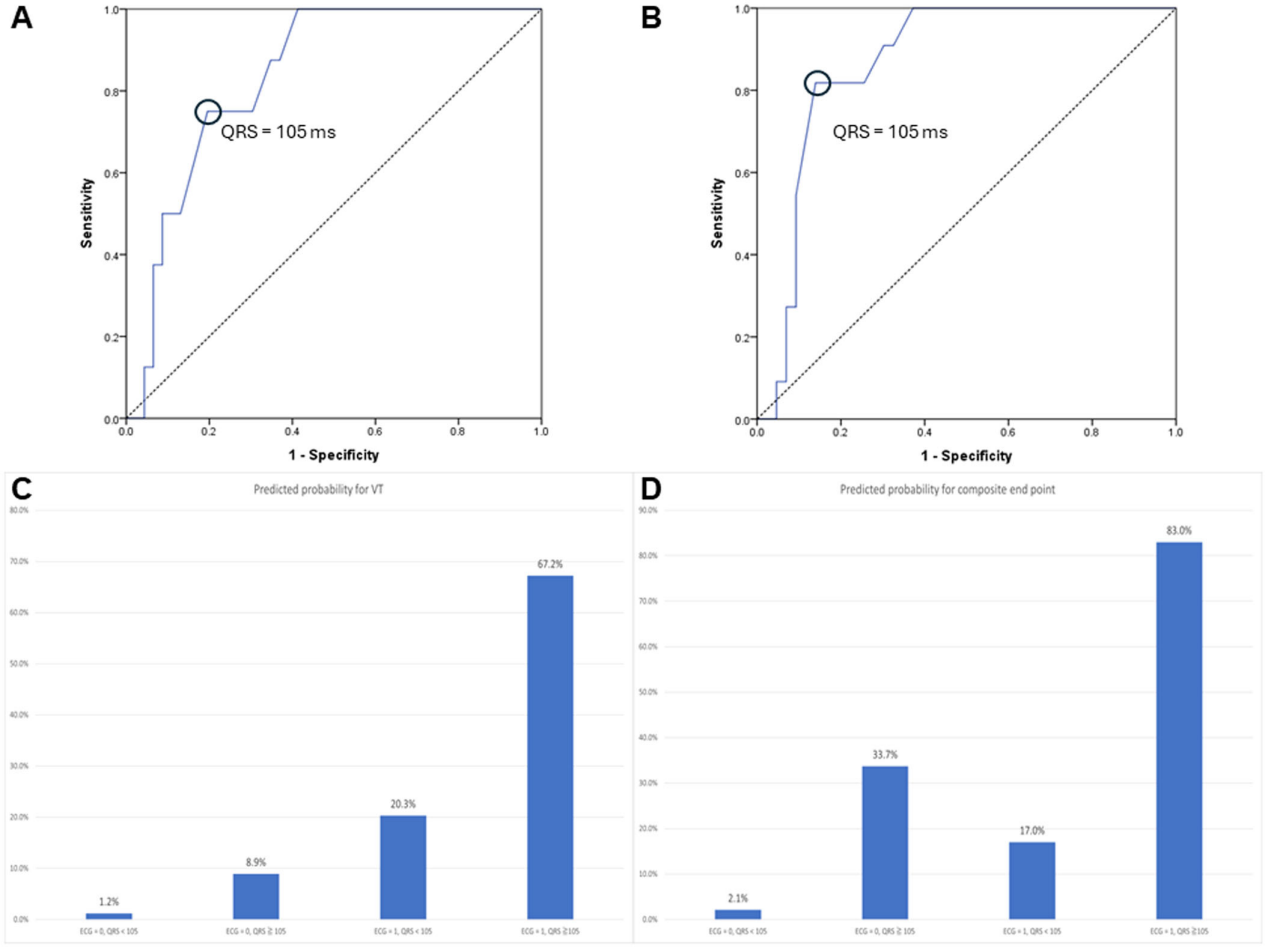
(A) receiver operating characteristic curve (ROC) of QRS interval to predict ventricular tachyarrythmia; (B) ROC of QRS interval to predict inhospital composite endpoint; (C, D) the predicted probability for ventricular tachyarrhythmia (C) and composite end‐point (D) using critical ECG and QRS interval. (ECG = 1: critical ECG) ECG = 0; noncritical ECG). ms, msec.

The ROC curve calculated the best cut‐off value of QRS width to be 105 ms for both VT (Figure 2A. AUC = 0.837, *p* = 0.003) and composite end‐point (Figure 2B. AUC = 0.869, *p* < 0.001).

Among the 11 patients with in‐hospital composite end‐points, eight had both critical ECG and prolonged QRS, three presented with noncritical ECG and prolong QRS, and two had critical ECG pattern without prolong QRS.

Additional analysis with logistic regression with covariates of ECG and QRS width revealed that the high probability of composite end‐point was 83% in patients with critical ECG and QRS width ≥ 105 msec (Figure 2D). It also revealed that the high probability of VT was 67.2% in patients with critical ECG and QRS width ≥ 105 msec (Figure 2C).

The predicted probability (p) for the composite end‐point was calculated using the propensity score method as follows,

ln(p/(1−p))=−7.205+0.046×QRSinterval+2.612×criticalECG

## 4 Discussion

This study highlights the importance of critical ECG patterns and prolonged QRS interval in predicting VT and in‐hospital mortality. Previous studies have demonstrated an association between lambda‐wave or tombstoning‐ST elevation. malignant ventricular arrhythmias and cardiac death in patients with MI [2, 3, 5, 11, 12]. However, this study adds to the existing literature by showing that the combination of critical ECG patterns and prolonged QRS intervals has additive predictive power for VT and in‐hospital death.

Critical ECG types were caused by large myocardial damage. A few studies have reported that lambda‐wave ST elevation is strongly associated with VT and cardiogenic shock in Takotsubo syndrome [13, 14]. Takotsubo syndrome with tombstoning‐ST elevation was presented as seizure with pulseless electrical activity [15, 16]. The critical ECG characteristics had similar bad outcomes in Takotsubo syndrome as myocardial infarction.

Additionally, Takotsubo patients with a prolonged QRS interval of ≥ 120 ms have higher in‐hospital mortality and cardiac death compared to those with a QRS interval < 120 ms [17]. Laurence et al. found that life‐threatening arrhythmias could be predicted by independent factors of lower left ventricular ejection fraction and QRS > 105 ms in Takotsubo cardiomyopathy [18]. The prolong QRS interval indicates severe myocardial ischemia‐related Purkinje‐ventricular conduction delay [19, 20]. The Purkinje system, which is involved in ventricular conduction, may contribute to the re‐entry circuit in patients with post‐infarction monomorphic VT [21].

Overall, this study emphasizes the significance of critical ECG patterns and prolonged QRS interval in predicting VT and in‐hospital death, particularly in the context of Takotsubo syndrome.

### 4.1 Incidence of the Lambda‐Like and Tombstone Pattern

The incidence of lambda‐like ST elevation in Takotsubo syndrome has been reported to be 3.2% [14]. This specific ST elevation pattern was strongly associated with in‐hospital complications such as cardiogenic shock or death. It was found to be indicative of high‐risk hemodynamics in patients with Takotsubo syndrome, as it reflected transient severe left ventricular dysfunction rather than myocardial ischemia.

In previous studies, tombstone ST elevation was shown to be similar to the lambda‐like ST elevation in case reports of Takotsubo syndrome. These un‐differential figures were described as tombstone ST elevations in Takotsubo syndrome based on case reports [15, 16]. But no original study has reported its incidence in patients with Takotsubo disease. Tombstone ST elevation has been observed in 10%–26.1% of patients with ST‐elevation myocardial infarction (STEMI), and has been shown to increase mortality and the risk of ventricular arrhythmias in patients with acute myocardial infarction [3].

In this study, the incidence of critical ECG was higher than in above references. The lambda‐like ECG pattern was observed in 16.3% (9/55 patients) and the tombstone‐like ECG pattern was observed in 12.7% (7/55 patients) of Takotsubo patients. These two critical ECG patterns occurred during the first 3 days of admission. The incidence was 5.5% for lambda‐wave ST elevation (3/55 patients) and 7.2% for tombstoning ST elevation (4/55 patients) on the first day of visiting the emergency room. Under continuous monitoring, there were 5 patients with critical ECG patterns occurring on the second day of admission and the remaining 4 patients with critical ECG patterns occurring on the third day of admission. The first‐day incidence of critical ECG was close to other studies. However, this retrospective observation truly revealed a high prevalence of lambda‐wave ST elevation and tombstoning ST elevation, correlating with in‐hospital VT and composite events.

### 4.2 Mechanism

The release of catecholamines [22] and endothelin [23] can also lead to increased sympathetic activity and vasoconstriction in the coronary arteries, further exacerbating microvascular constriction and ischemia in Takotsubo syndrome.

Furthermore, regional myocardial edema and reduced myocardial contraction caused by sympathetic activation and catecholamine release can result in the release of N‐terminal proBNP, a myocardial injury marker [24].

Animal models have shown that local myocardial stretch can activate ion‐channels, including adenosine triphosphate (ATP)‐sensitive potassium channels, which can further alter the transmembrane potential and contribute to ST elevation [25].

The combination of microvascular ischemia, myocardial stretch, and altered transmembrane potential can lead to significant ST elevation in Takotsubo syndrome. This ST elevation often has severe convexity, that can merge with the R wave on an electrocardiogram to create a tombstone‐like pattern. In extreme cases, a lambda‐wave patterns, characterized by an extreme convex‐type pattern, can also be observed [14].

Cardiac magnetic resonance imaging revealed myocardial edema without scar formation in Takotsubo cardiomyopathy [24, 26]. This edematous area may contribute to reversible electrical alterations, leading to QRS blocking and QT prolongation. These electrical changes may increase the risk of functional reentry with monomorphic VT.

Reentry may have been the major mechanism for inducing monomorphic VT in our study group, similar to structure heart disease [27]. In this study, all eight patients initially presented with monomorphic VT, and one patient subsequently developed ventricular fibrillation (VF). The prevalence of VT in this study group was 13.5%, which is consistent with previous reports of life‐threatening ventricular arrhythmias in TTS [28]. Myocardial edema has also been correlated with repolarization abnormalities, which could potentially trigger polymorphic VT and torsade de pointes.

Abnormal conduction in the heart caused by myocardial edema, sympathetic activity, and catecholamines can prolong the QT interval [27, 29].

This can also result in QRS prolongation and atrio‐ventricular block, and not just affect the QT interval [17, 18, 30].

In our study, the patients with QT prolongation did not show significant differences between the VT‐group and non‐VT group. However, we found that QRS prolongation (≥ 105 ms) was an independent predictor for VT in this study. The presence of critical ECG abnormalities and a prolonged QRS duration indicates a more severe form of myocardial ischemia and edema in Takotsubo syndrome.

### 4.3 Study Limitations

The study had several limitations. First, this was a retrospective study conducted at a single center. Data collection relied on medical records and some VT episodes were only recorded using ECG strips in the intensive care unit, which may have led to inaccuracies in identifying and assessing VT episodes. Some patients were lost to follow‐up after discharge, which might have affect have the completeness of the data. This study did not explore other potential predictors of long‐term outcomes such as critical ECG types and QRS prolongation, which may have provided further insights.

Second, LVEF was measured by different doctors, and interobserver variation existed and could not be modified. A significant reduction in LVEF is reasonable in severe myocardial ischemia; however, the LVEF was not significantly different between the VT and non‐VT groups.

Third, several factors including LVEF, J point elevation, and sum of ST deviations, were not statistically significant because of the small number of patients enrolled. Additionally, the BNP levels were not completely assessed in all patients. In our hospital, N‐terminal pro‐B‐type natriuretic peptide has been used instead of BNP in recent years. All these factors had an impact on the clinical outcome of TC by previous studies [31, 32].

Additionally, physical stress could be clearly documented by reviewing medical records, but emotional stress could not be documented owing to incompletely recordings. Most patients with uncertain stress are suspected of experiencing emotional stress.

## 5 Conclusion

The results of this retrospective study indicated a significant occurrence of ventricular arrhythmia and in‐hospital death, with a rate of 21.1%.

The study also identified two critical ECG types, tombstoning ST elevation and lambda‐wave ST elevation, which had a strong impact on short‐term outcomes. These ECG findings can potentially serve as early warning signs for the development of complications and should be closely monitored.

Furthermore, the presence of conduction disturbance with a prolonged QRS interval of ≥ 105 ms was found to be a independent predictor for in‐hospital complications.

Overall, these findings highlight the importance of identifying high‐risk patients and implementing close monitoring and aggressive interventions to improve the outcomes in this subgroup of patients.

## Author Contributions

**Jen‐Te Hsu:** data analysis, drafting article. **Ju‐Feng Hsiao:** design, critical revision of article. **See‐Khong Chin:** design, data collection. **Yu‐Cheng Hsu:** design, data collection. **Meng‐Huan Lei:** concept, approval of article.

## Ethics Statement

The study was approved by the local ethical committee. (IRB112‐159‐B) from the Research Ethics Committee of Hualien Tzu Chi Hospital, Buddhist Tzu Chi Medical Foundation.

## Conflicts of Interest

The authors declare no conflicts of interest.

## Data Availability

The data that support the findings of this study are available from the corresponding author upon reasonable request.
